# Obstetricians’ perceptions of midwife specialists’ roles in South Africa

**DOI:** 10.4102/phcfm.v18i1.5276

**Published:** 2026-04-17

**Authors:** Kagiso P. Tukisi, Zelda Janse van Rensburg, Wanda Jacobs

**Affiliations:** 1 Department of Nursing, Faculty of Health Sciences, University of Johannesburg, Johannesburg, South Africa

**Keywords:** obstetricians, midwife specialists, scope of practice, medical litigations, defensive practice

## Abstract

**Background:**

The midwife specialists (MS) are trained midwifery professionals with advanced knowledge and skills to perform interventions to respond to complicated maternal and neonatal conditions. A midwife specialist is expected to function independently and interdependently with other healthcare professionals such as obstetricians and paediatricians. However, studies have shown that the circumstances within public hospitals could be more favourable to the midwife specialist’s independent and autonomous practice.

**Aim:**

This study aims to explore and describe obstetricians’ perceptions of midwife specialists‘ roles in public hospitals in South Africa.

**Setting:**

The study was conducted in the selected public hospitals in South Africa.

**Methods:**

Authors followed a qualitative, descriptive, explorative research design. Data collection took place between March 2022 and June 2022. Purposive sampling was utilised to sample nine obstetricians to participate in online individual semi-structured interviews. Data were analysed using Colaizzi’s seven-step method.

**Results:**

Three themes emerged. In theme 1, midwife specialists are skilled professionals practicing with limitations. In theme 2, there is an absence of the specific scope of practice (SOP) for midwife specialists’. In theme 3, they are defensive practitioners with over-reliance on physicians and clinical records.

**Conclusion:**

The SOP should be revised to grant midwife specialists full professional and legal authority to practice independently. Removing these limitations would enable them to collaborate effectively with physicians, ensuring safe and comprehensive maternal and neonatal care.

**Contribution:**

This study highlights the state of midwife specialists practice in the public hospitals of South Africa. Obstetricians elucidate various barriers to midwife specialists autonomous and collaborative midwifery care.

## Introduction

In South Africa, the midwifery profession is divided into two categories based on training and registration with the South African Nursing Council (SANC).^1^ There are registered midwives and registered midwife specialists (MS).^1^ A registered midwife is a healthcare professional who is trained in a 4-year bachelor of comprehensive nursing and midwifery programme or a 3-year nursing programme with an additional 1-year midwifery training. These trainings lead to the registration as nurses and midwives.^2^ The registered midwives are qualified to provide low-risk comprehensive prenatal, antenatal, intrapartum, neonatal and postnatal care.^3^ A midwife specialist holds a postgraduate diploma or degree in midwifery. Therefore, a midwife specialist has a more advanced knowledge and skill set. The midwife specialists are responsible for the management of high-risk antenatal, intrapartum, neonatal and postnatal care.^3^ The midwife specialists are part of the interprofessional collaborative team with the obstetricians for better maternal and neonatal outcomes.^4^

The collaborative relationship between the midwife and the obstetrician is one of the long-standing interprofessional relationships in the health science sphere.^5^ The obstetricians are predominantly responsible for managing complicated pregnancy, labour and puerperium.^6^ The obstetrician’s handling of the complications aligns with their extensive and prolonged training and the broader scope of practice (SOP).^7^ The midwives, on the other hand, are responsible for managing low-risk pregnancies, labour, puerperium and newborn.^8^ The midwives’ function in a low-risk context is aligned with their training and the SOP that prescribed parameters for low-risk midwifery.^8^

While midwives play an integral part in maternity healthcare services, their limited skill set and knowledge can be insufficient to enable them to respond to patients’ needs and prevent deleterious outcomes.^1^ Midwives are often left to their own devices with high-risk patients because of the shortage of obstetricians to handle such cases, mainly in the clinical facilities located in rural areas.^1^ Consequently, a resolution was taken by the Department of Health and the SANC to expand the knowledge and skill set of the midwives through midwife specialists’ training.^9^

A midwife specialist is a midwife who has undergone postgraduate midwifery training under the phased-out education and training regulations 212, 1993 and under new curriculum 635, 2020 of the SANC, leading to registration as a midwife specialist.^10,11^ Midwife specialist training equips the candidate with specialised midwifery knowledge and skills to perform life-saving interventions on complicated patients in need of maternity health services.^12^ The expansion of midwives’ knowledge and skills is not a new phenomenon; South Africa is one of the countries that have invested in the midwives’ knowledge and skills to respond to the dire needs of the patients seeking maternity healthcare services.^6,12^

The midwife specialists are better trained than registered midwives to handle complicated patients without the doctors and in collaboration with the doctors.^9^ The South African health system depends on the patients’ referral system from less specialised to more specialised levels of care.^12,13^ This mode of healthcare service places the midwives and midwife specialists at the forefront of initiating the maternity healthcare service.^14^ The placement of registered midwives and registered midwife specialists at the forefront aligns with the midwifery-led models of care, which seek to grant midwives and midwife specialists discretionary and autonomous powers to lead in patients’ care.^15,16^

While there is great expectation for midwife specialists to contribute immensely to patient care, the midwife specialists are unable to optimally utilise their knowledge and skills set.^4,17^ Midwife specialists work hand in hand with the doctors and obstetricians in a multidisciplinary team.^11,18^ This study is part of a doctoral study where the optimal utilisation of midwife specialists’ knowledge and skills was explored in its entirety from the multivariate population. Therefore, this part of the study sought to explore and describe the roles of midwife specialists through the lens of obstetricians.

## Research methods and design

A qualitative, exploratory and descriptive research design was followed to explore and describe the midwife specialist’ roles in South Africa through the lens of obstetricians.^18^ The obstetricians work directly and collaboratively with the midwife specialists and are therefore knowledgeable about their practice. The explorative and descriptive research design was relevant to uncovering information on the phenomenon under study as perceived by the obstetricians in South Africa.^18^ The explorative research enabled the obstetricians to bring forth their own perceptions of the midwife specialists’ roles in the public hospitals.^18^ The descriptive research design enabled the obstetricians to paint a picture of registered midwife specialists’ practice as it occurs naturally in the public hospital in South Africa.^18^

### Study’s setting

The study took place in the public regional and academic hospitals within the six provinces of South Africa. The public regional and academic hospitals are pivotal in providing essential maternal and neonatal healthcare services to 70% of South African citizens.^19^ Most of the midwife specialists are employed by the public health sector and work collaboratively with the obstetricians.^19^

### Population

The population comprised the obstetricians employed by the academic hospitals in South Africa. An academic hospital is a higher level of care where high-risk maternal and neonatal clients with complex obstetrical and neonatal conditions receive specialised healthcare.

### Sampling strategy

The study is qualitative, explorative and descriptive and not categorised as a clinical trial.^20^ Therefore, a clinical trial number is not applicable. Further approval was obtained from the National Department of Health and provincial Department of Health to conduct health science-related research. A purposive sampling was used to recruit the obstetricians via the clinical managers who acted as gatekeepers.^21^ The gatekeepers introduced the researcher to the participants and shared the research information letters. Interested participants made telephone contact with the researcher, and the email addresses were shared.^21^ The researcher made it a point to keep the participant’s contact details confidential and restricted communication of research information in line with the *Protection of Personal Information Act* (*POPIA Act 4 of 2013*).^23^ The following inclusion criteria were used to select the participants:

### Inclusion criteria

The study included participants who met all of the following criteria:

- Registered medical doctors with a bachelor’s degree in medicine (MBCHB).
- A master’s degree (MMed) in Obstetrics and Gynaecology, registered with the Health Professions Council of South Africa (HPCSA).
- A minimum of 2 years of experience as obstetricians in public health sectors in South Africa.

### Exclusion criteria

Registered medical doctors with a bachelor’s degree in medicine (MBCHB) and a master’s degree (MMed) in Obstetrics and Gynaecology, registered with the Health Professions Council of South Africa (HPCSA) were excluded from participation if they:

- Had less than 2 years of professional experience as obstetricians.
- Were not currently employed in public academic hospitals in South Africa.

A total of 9 obstetricians from six academic hospitals located in six provinces of South Africa participated in the individual semi-structured interviews. The participants were recruited from the following six provinces, respectively, North West, Gauteng, Limpopo, Free State, Eastern Cape and KwaZulu-Natal. All the participants met the inclusion criteria. The selected sample’s clinical experience in the public and private sectors of South Africa was between 10 years and 23 years at the time of data collection. The participants’ demographic data are summarised in Table 1.

**TABLE 1:** Participants’ demographic data.

Participants’ code: Obstetrician	Qualifications	Years of experience	Facility	Province
1	Bachelor of Medicine and Surgery Master’s in Medicine: Obstetrics and Gynaecology	17	Academic hospital	North West
2	Bachelor of Medicine and Surgery Master’s in Medicine: Obstetrics and Gynaecology	13	Academic hospital	Gauteng
3	Bachelor of Medicine and Surgery Master’s in Medicine: Obstetrics and Gynaecology	16	Academic hospital	Limpopo
4	Bachelor of Medicine and Surgery Master’s in Medicine: Obstetrics and Gynaecology	10	Academic hospital	Free State
5	Bachelor of Medicine and Surgery Master’s in Medicine: Obstetrics and Gynaecology	18	Academic hospital	Eastern Cape
6	Bachelor of Medicine and Surgery Master’s in Medicine: Obstetrics and Gynaecology	23	Academic hospital	KwaZulu-Natal
7	Bachelor of Medicine and Surgery Master’s in Medicine: Obstetrics and Gynaecology	21	Academic hospital	Gauteng
8	Bachelor of Medicine and Surgery Master’s in Medicine: Obstetrics and Gynaecology	17	Academic hospital	Eastern Cape
9	Bachelor of Medicine and Surgery Master’s in Medicine: Obstetrics and Gynaecology	15	Academic hospital	Gauteng

### Data collection

Data collection took place between March 2022 and June 2022. At the time of data collection the first author was employed as a midwife specialist and a midwifery and neonatal nursing lecturer at the health sciences university in South Africa. Before the data collection approval from the University of Johannesburg Research Ethics Committee (UJREC), the provincial health department and respective hospitals were sought. The chief executive officers and clinical managers provided permission to access the obstetricians.^21^ The researcher presented the study in the maternal and neonatal morbidity and mortality meetings to provide the obstetricians with information pertaining to the study. The obstetricians who were interested in participating in the study contacted the authors’ using the contact details in the research information letter. Appointments were arranged at the convenience of the participants. The authors were cognisant of the obstetricians’ demanding responsibilities and obligation to maternal and neonatal care. A total of 17 obstetricians were recruited to participate in the study. During the interactions with the prospective participants the authors requested email addresses that were used to circulate the links for Microsoft Teams scheduled at the date and time convenient for each participant. A total of 9 obstetricians attended the online interviews and eight were unable to honour the invitations because of work emergencies and obligations.

The participants were informed that the semi-structured interview will be conducted on Microsoft Teams and a signed consent for their participation in the study was obtained through email.^21^ The participants were conversant with the Microsoft Teams as it was a meeting platform for the meetings and webinars during COVID-19 pandemic. The participants were informed that the interview will be audio recorded; however, the video camera function of the Microsoft Teams was disabled to ensure privacy and confidentiality.^22^ A signed consent for recording of the interview was obtained via the email.

The individual interviews lasted between 60 min and 90 min. An interview guide comprising a central question and probes was derived from the research objective. One central question was asked of all participants: *As an obstetrician, what is your perception of midwife specialist role in South Africa?*

The probes followed up on the central question to uncover midwife specialists’ practice in-depth. In addition, probing questions were used to direct the flow of conversations and gain in-depth information from the participants regarding the phenomenon under study.^21^

Despite the virtual nature of the Microsoft Teams platform and participants’ cameras being turned off the authors collected field notes. The researcher carefully listened to the participants’ vocal intonations, emphasis, sighs and prolonged pauses to complement the verbal content of the participants’ responses.^20^ The first two interviews were regarded as a pilot interviews to assess the comprehensibility of the central question.^21^ The pilot interviews yielded data consistent with data from subsequent individual interviews. Consequently, data from the pilot study were included in the data from the main sample.^20^ Data saturation was reached during the sixth interview when no new data emerged, and three more interviews were conducted to confirm saturation.

### Data analysis

Data obtained from the individual semi-structured interviews was transcribed using Microsoft Team TM automated voice transcriptions. However, the authors validated the accuracy of the transcriptions by listening to the audio recording against transcriptions. Data were analysed using Collaizi’s seven steps of qualitative data analysis.^22^ Following verbatim transcription, the authors listened to the recordings and read the transcriptions several times to gain an understanding of the obstetricians’ perceptions of the midwife specialists’ practice. The authors’ immersion in data led to the authors extracting significant statements relating to midwife specialists’ practice. The authors reviewed each interview and extracted the statements, which relate directly to the midwife specialists’ practice. The authors analysed the transcriptions to interpret and uncover the hidden meanings underlying the statements from the obstetricians’ perceptions of the midwife specialists’ practice. The significant statements were clustered into categories and themes detailing the midwife specialists’ practice in the public health sectors of South Africa. The formulated meanings were then organised into themes detailing the midwife specialists’ practice as perceived by obstetricians. The results of the study on midwife specialists’ practice were integrated to create clusters and themes to form full descriptions of obstetricians’ perceptions of midwife specialists’ practice. The theme clusters and themes that emerged from the formulated meanings were integrated to form an exhaustive description of the obstetricians’ perceptions of midwife specialists’ practice. At the end of each interview, the first author summarised each interview to the participants to validate that the participants’ intended meanings were captured. The independent coder was employed to identify themes. To ensure confidentiality and anonymity of the research participants, an independent coder signed a non-disclosure agreement. A consensus meeting was held between first author and an independent coder to finalise the themes.

### Trustworthiness

Lincoln and Guba’s 1985 strategies were followed to increase the trustworthiness of the study.^24,25^ The strategy of credibility, transferability, dependability, confirmability, and authenticity were applied as follows: To increase credibility of the study, the authors spend time with the participants during the recruitment process by presenting the research information letter during the maternal and neonatal morbidity and mortality meetings.^24,25^ In addition, the researcher allowed communication with the participants to determine the duration of the interview. Therefore, the interviews were not rushed, and the participants had an opportunity to express themselves without interruptions and with in-depth engagements during the interview.^24,25^ The participants’ vocal cues such as prolonged sighs and slow speaking pace were observed for their underlying meanings. To ensure transferability, the researcher described South Africa’s public health sector where midwife specialists practice collaboratively with the obstetricians. In addition, the researcher clearly described the participants’ demographic data to bring the context to the reader’s attention. Dependability of the study was ensured by collecting data up to a point of data saturation.^21^ An independent coder was employed to eliminate potential bias in data analysis. Confirmability was ensured by bracketing the authors’ preconceived ideas and reporting the participants’ own perceptions of the midwife specialist’s practice. The first author as midwife specialists documented his preconceived idea that the obstetricians did not perceive midwife specialists as collaborators within maternal and neonatal care context and approached the field with an open mind. Additionally, the researcher documented all the information pertaining to the research process. To increase the authenticity of the study the researcher captured the participants’ voices verbatim, which were used to derive themes and subthemes. Furthermore, the themes and subthemes were supported by variety of quotations from the participants, which ensured diversity.^24,25^

### Ethical considerations

The research involves human participants that warrants adherence to health science research ethics detailed in Helsinki Declaration.^23^ The University of Johannesburg Research Ethics Committee and Higher Degrees Committee (REC-1279-2021; HDC-01-154- 2021) granted permission to conduct the study. Further approval was obtained from the seven provinces and their respective academic hospitals. The authors selected participants using the selection criteria in the approved research proposal, which ensured fairness; thus, the principle of justice was upheld.^23^ The research information letter was presented to the prospective participants to enable them to make informed decisions about their participation in the study.^21^ The participants were informed about their prerogative to withdraw from the study without any punishment, which ensured their autonomy.^20^ To maintain the participants’ privacy and confidentiality, the individual semi-structured interviews were conducted on Microsoft Teams, and video camera was disabled.^22^ Alphanumeric codes were generated for discussion of data to ensure participants’ anonymity. All the documents and recordings were kept in password-encrypted files only known to the authors for 5 years. There were no anticipated harmful effects associated with the participation in the study. However, the obstetricians reflected in the midwife specialists’ practice, which could lead to better collaborations between them and midwife specialists for better patients’ outcomes.

## Results

Three themes with subthemes emerged, as detailed in Table 2. A central paradox of these themes is that the midwife specialists possess specialised clinical skills. However, they are constrained by the unclear regulatory and professional boundaries. Theme 1 elucidates that midwife specialists are knowledgeable and skilled professionals who lack professional autonomy in their specialist’s role. Theme 2 highlights that the midwife specialists practice constraints are associated with the absence of a specific SOP to guide their specialist’s role. Theme 3 highlights that the midwife specialists’ poorly defined roles and susceptibility to medical litigations cascaded to defensive midwifery practice.

**TABLE 2:** The summary of the data analysed.

Themes	Subthemes
1. Midwife specialists are knowledgeable and skilled but practice with limitations	1.1. Midwife specialists’ knowledge and skills 1.2. Midwife specialists’ limited practice
2. Absence of specific scope practice of midwife specialists	2.1. Undefined roles and responsibilities 2.2. Unclear distinction between registered midwives and midwife specialists 2.3. Midwife specialists’ susceptibility to medical litigations
3. Midwife specialists are defensive practitioners	3.1. Overdependence on physicians 3.2. Record-keeping as a defence tool

### Theme 1: Midwife specialists are knowledgeable and skilled but practice with limitations

Under this theme the obstetricians verbalised their awareness of midwife specialists as knowledgeable and skilled professionals. However, they were concerned about the midwife specialist lack of autonomy in clinical practice.

#### Subtheme 1.1: Midwife specialists’ knowledge and skills

The obstetricians affirmed that the midwife specialist were knowledgeable and skilled professionals who may contribute immensely to patients’ care. The obstetricians based their affirmations on their awareness of midwife specialist to provide clinical teaching in Medical Schools in South Africa:

‘I mean, I was at Wits [*University of the Witwatersrand*], and my friends and colleagues from S.M.U. [*Sefako Makgatho Health Sciences University*] and UOFS [*University of Free State*] can attest to this. Our lecturers included all the multidisciplinary team members, including nurses and midwife specialists. It was worse in clinical because 80% of our clinical lecturers were Specialized nurses.’ (Obstetrician 1, 17 years’ experience, North West)

‘We are not meeting advanced midwives as colleagues in the wards and training for the first time. Advanced midwives are part of the teaching staff, even in universities. I was a clinical facilitator for MEDUNSA [*Medical University of Southern Africa*], and we had sisters [*Midwife specialists*] teaching clinical.’ (Obstetrician 3, 16 years’ experience, Limpopo)

Obstetricians highlighted that the midwife specialist supported them in the clinical facilities as junior doctors at the start of their medical profession. Therefore, obstetricians had confidence in midwives specialist knowledge and skills:

‘I have absolute trust and respect for midwife specialists. I was taught how to deliver babies by the advanced midwives back in varsity. I am an obstetrician, and I am today because of their contributions. Midwife specialists are knowledgeable and skilled.’ (Obstetrician 2, 13 years’ experience, Gauteng)

‘I was taught to deliver a breech by the advanced midwife as an intern [*physician*]. She called me for a breech presentation, and I was in panic mode trying to reach my senior, who was in theatre at the time, but then the patient was delivering.’ (Obstetrician 7, 21 years’ experience, Gauteng)

#### Subtheme 1.2: Midwife specialists’ limited practice

Although MS was undoubtedly knowledgeable and skilled, the obstetricians highlighted that the midwife specialist was practicing with limitations. The limited practice of midwife specialist was linked to the level of care of the facility where they worked. The higher levels of care facilities, such as academic hospitals, render the doctor care, limiting the midwife specialist’s autonomous practice:

‘The midwife specialists here know how to manage the woman but must wait for the doctor to prescribe it. That limits them. However, then there are differences between these levels of care, and with an increase in the level, so is the risk, and that is why more doctors, senior doctors, and specialists take the lead.’ (Obstetrician 5, 18 years’ experience, Eastern Cape)

### Theme 2: Absence of specific scope practice of midwife specialists

Obstetricians highlighted that while they are aware of midwife specialists’ knowledge and skills, there is no SOP that specifies the midwife specialist’s roles and responsibilities. They echoed that it is challenging to determine their specific roles and responsibilities in the clinical facilities. The presence of registered midwives in clinical facilities exacerbated the challenge as the obstetricians were unable to distinguish between the categories of midwives. In addition, the midwife specialists are litigation prone.

#### Subtheme 2.1: Undefined roles and responsibilities

The obstetricians expressed that the midwife specialists’ roles and responsibilities must be better defined in the clinical practice. The obstetricians echoed that although the MS were specialists in knowledge and skills, their roles needed to be integrated effectively into the health system. Consequently, the midwife specialists were unable to practice as specialists:

‘Midwife specialists need to be adequately integrated into the system and the practice so they can assume their roles and responsibilities as specialists. So, we can safely say that although the advanced midwives are knowledgeable and we know that they are, there are no prescribed roles for them in the hospitals.’ (Obstetrician 4, 10 years’ experience, Free State)

They echoed that well-defined roles and responsibilities were important for interprofessional collaboration (IPC) because as obstetricians they needed to understand the midwife specialists’ professional practice and contributions. They highlight a need for the midwife specialists’ roles and responsibilities to be redefined:

‘We are doing interprofessional collaborations in our universities where health professions students learn a lot about what other colleagues are doing. So, you can already see that we need roles to be well-defined moving forward. So, I am going back to the council, and there must be roles to ensure that advanced midwifery [*old concept used to refer to midwife specialist*] is standardized. So, if we all know the role of advanced midwives, that could influence policy formulation.’ (Obstetrician 6, 23 years’ experience, KwaZulu-Natal)

#### Subtheme 2.2: Unclear distinction between registered midwives and midwife specialists

The obstetricians were aware of the registered midwives and midwife specialists as categories in the midwifery profession. However, they had challenges differentiating between the registered midwives and midwife specialists:

‘There is a real challenge there: identification is a problem. As doctors, we must find out who a midwife or specialist is. To most doctors, a midwife is a midwife or a sister working in a maternity ward is a midwife. So, we need to find out who a midwife specialist is.’ (Obstetrician 9, 15 years’ experience, Gauteng)

The obstetricians’ challenge in identifying midwife specialists was linked to the absence of well-defined roles and responsibilities, which resulted in similarities in the practice of both the registered midwives and midwife specialists in clinical practice:

‘To us [*obstetricians*], they are all midwives, because they are working exactly the same way. It is probably because they are adhering to the same scope of practice.’ (Obstetrician 3, 13 years’ experience, Limpopo)

#### Subtheme 2.3: Midwife specialists’ susceptibility to medical litigations

The obstetricians acknowledged that the absence of a specific SOP was the main reason for midwife specialists’ limited practice in the clinical facilities. The limited practice was out of fear of potential litigations. The absence of SOP suggested that the midwife specialist’ practice might not be fully legalised by South Africa, which made the midwife specialists susceptible to litigation:

‘A council [*South African Nursing Council*] here regulates the practice, so it starts there. Is the council allowing midwife specialists to be specialists? Do they have legal authority to treat patients using a specialist’s cap? Moreover, that is precisely what they fear! They are knowledgeable and skilled, but what we are picking up is that something extensive stands in their way. The only thing that can unsettle a professional like this is going against professional conduct because you will be barred from practicing.’ (Obstetrician 8, 17 years’ experience, Eastern Cape)

The obstetricians sympathised with the midwife specialists, as the patients and family members, who are inquisitive in every intervention, raised susceptibility to litigations:

‘On the other hand, our communities need to make practice easier for professionals because they always want positive outcomes. We are in the health sciences, and our interventions will only sometimes be successful, although we are looking at a 100% success rate. So, every patient we see is a potential risk who can sink one’s career. That might be why they do not want to practice if they are not legally allowed to do that.’ (Obstetrician 4, 10 years’ experience, Free State)

### Theme 3: Midwife specialists are defensive practitioners

Obstetricians were troubled by the midwife specialists’ defensive approaches to midwifery care. They were overdependent on the obstetrician’s contrary to an expectation that midwife specialists are knowledgeable and skilled professionals. Furthermore, the midwife specialists use clinical records as defence tool.

#### Theme 3.1: Overdependence on physicians

The MS came across as defensive in their practice. According to the obstetricians, midwife specialist seemed to rely more heavily on the doctors to avoid possible involvement in litigations. The MS’s over-reliance on doctors contravened the obstetricians’ expectations:

‘I have noted that nurses [*Midwife specialists and other clinical nurse specialists*], even with their specialist courses, cannot move away from dependence on doctors. So, you find that orders are received and carried out without queries or discussions. Doctors will sometimes feel like they cannot be told what to do! However, it is not like someone must dictate; we are having a professional clinical discussion.’ (Obstetrician 5, 18 years’ experience, Eastern Cape)

The obstetricians explained that some of the midwife specialists preferred to skilfully perform some life-saving interventions in the presence of obstetricians and sometimes even offered their assistance. Such instances left obstetricians questioning the reasons underlying midwife specialists’ dependent roles:

‘They can call you for a complicated delivery, say there is this very breech delivery, and you get there but struggle with it. The very same person who called you for the case will ask you to step aside so that she can assist, and believe me, she will get that baby out. This is a usual practice in our facilities, and then you ask yourself, did the sister have to call for my help.’ (Obstetrician 1, 17 years’ experience, North West)

The MS overdependence was associated with the absence of the relevant SOP. Consequently, midwife specialists were reluctant to perform interventions they were knowledgeable and skilled to perform and resorted to calling the doctors to assist in protecting themselves from possible litigations:

‘So, for things that they can do and know are beyond their scope of practice e or not covered by policies, they will always call the doctors for it.’ (Obstetrician 2, 13 years’ experience, Gauteng)

‘They are aware that the scope of doctors is broad, and most procedures fall under them, so they will always seek protection from doctors.’ (Obstetrician 4, 10 years’ experience, Free State)

The MS’s dependence on the doctors left the obstetricians inundated with patients, as the midwife specialists were unable to assist with performing some of the interventions. Consequently, the obstetricians were frustrated and overwhelmed with the patients’ care responsibilities:

‘So, you can already see that we are very defensive in our practice. So, we must acknowledge that even advanced midwives are professionals and answerable to their council. The doctor, on the other hand, feels overwhelmed. That is why I am doing everything when I am supposed to be supported by the team.’ (Obstetrician 1, 17 years’ experience, North West)

#### Theme 3.2: Record-keeping as a defence tool

The obstetricians highlighted that under certain circumstances midwife specialists are required to perform some of the advanced interventions. However, they insist on the presence of a physician and documentation to that effect. Obstetricians concluded that the midwife specialists seek to protect themselves from litigations:

‘The sister calmly delivered the woman and asked me to write the notes that the doctor was called so that it could be documented. I only realized later that the sister was trying to protect us all, which puzzled me because she knew exactly what she was doing.’ (Obstetrician 5, 18 years’ experience, Eastern Cape)

The obstetricians were also equally wary of their potential involvement in litigations. Therefore, the midwife specialists’ recording styles proved stressful for the obstetricians and their subordinates, as they felt that the midwife specialists were opening them up for litigation:

‘Doctors are also scared of potential litigations and find it very stressful when the sisters have documented their names in the records, especially in cases of adverse perinatal outcomes.’ (Obstetrician1, 17 years’ experience, North West)

The obstetricians pointed out that midwife specialists worked with junior doctors and sometimes had to step in and perform advanced obstetric interventions during emergencies. However, the midwife specialists often required junior doctors to complete clinical records to confirm their presence and involvement in the procedures:

‘I have noted that although they can do all these advanced manoeuvres, they are weary of litigations because they will insist on the intern writing notes that they were called and were present at the delivery.’ (Obstetrician 3, 16 years’ experience, Limpopo)

## Discussion

The study aimed to explore and describe the South African MS knowledge and skills in the public sectors of South Africa through the lens of obstetricians. It became evident that the obstetricians believed that midwife specialists are knowledgeable and skilled professionals who can immensely contribute to the welfare of patients in obstetrics and gynaecology departments. This finding supports the main objective of the inception of the midwife specialists training programme in South Africa, which was aimed at increasing midwife specialists’ knowledge and skills to respond to the health needs of patients.^16^

Regrettably, despite the MS knowledge and skills, the midwife specialists seemed to need to improve their application of such knowledge and skill, which obstetricians described as limited practice. This finding is consistent with the studies conducted in Liberia and Uganda, where midwife specialists are empowered with advanced knowledge and skills in advanced midwifery.^5^ However, their professional autonomy remains a challenge.^14,17^ The midwife specialists’ limited practice was linked to the absence of SOP to provide legal protection during specialist practice. This finding is consistent with the studies recommending to the ministries of health and professional regulatory bodies to recognise the advanced knowledge and skills of midwife specialists.^5,26^

The obstetricians echoed that it was challenging to differentiate between the registered midwives who are non-specialised and the midwife specialists, which evidenced the existing hindered practice among the midwife specialists. This finding correlates with the midwife specialists’ self-perception of practice at the same level as registered midwives.^27^ The apparent similarities between the practice of registered and midwife specialists are based on the reality that they both function under the same SOP despite the knowledge and skills gap.^26^ In addition, this finding reveals the need for more definition of professional boundaries, roles, and responsibilities of registered midwives and midwives’ specialists, which could have been defined using distinct SOP and the International Confederation of Midwives (ICM) competencies of midwife specialists.^26^

Obstetricians were concerned about midwife specialists not stepping up to their professional roles, consequently leaving obstetricians inundated with caring responsibilities for patients. The obstetricians’ outcry for midwife specialists’ participation in patients’ care is a valid concern. According to Kaiser and colleagues, the midwife specialists and obstetricians form an IPC, which requires collective decision-making and action regarding patients’ care.^18,27^ Regrettably, the obstetricians experienced midwife specialists’ hindered practice as a form of professional overdependence as midwife specialists relied more heavily on the obstetricians. This finding is consistent with the midwife specialists’ experiences of over-reliance on obstetricians and less professional autonomy.^28^ This finding also contravenes the aim of developing the midwife specialists’ programme, which was aimed at strengthening midwife specialists’ independent and interdependent function.^29^

While the obstetricians found the IPC with the midwife specialists challenging because of the unclear roles and responsibilities, the obstetricians sympathised with the midwife specialists. The obstetricians explained that the midwife specialists evident hindered practice and overdependence on them, suggesting the generalised fear of litigation. The obstetricians’ claim is valid, considering an accelerated rate of litigations in maternity and neonatal cases. The South African Department of Health litigation bill amounts to R77 billion, and most claims are maternity and neonatal-related and include cerebral palsy and non-standardised procedures.^30^ The practice of partially legalised midwife specialists may land them in litigation, which may explain the midwife specialists’ defensive practice.

The midwife specialists’ defensive practice was evidenced in the manner of recording-keeping. The obstetricians expressed that the midwife specialists’ record keeping was a precise protective measure against potential litigations as the midwife made it a point to indicate the information about the obstetricians involved. This finding suggests that the midwife values record-keeping as a valuable tool for recounting the series of clinical events leading to adverse outcomes in the case of litigation.^31^ Additionally, it demonstrates the anxiety levels of midwife specialists during patient care. According to Thumm and the team, midwife specialists are anxiety prone as they must attend to obstetric complications and must make sound clinical decisions under stressful circumstances.^32^ The onset of obstetric emergencies causes fear and anxiety, the potential outcomes such as mortality, which could bring about a litigation case.^33^

### Strengths and limitations

Although the sample size is limited in the total number of participants, each participant is an expert and respected professional in obstetrics and gynaecology with a very close alliance with the midwife specialists in clinical practice. The authors planned to conduct a study in all nine provinces of South Africa. However, seven provinces approved the study. The scarcity of the obstetricians because of time constraints resulted in nine participants from six provinces. The small sample size and the number of provinces represented in the study suggest that the findings cannot be generalised to the South African context. On the other hand, the recruitment of participants from six provinces and hospitals demonstrates geographical diversity. Therefore, the study was not isolated to a single geographical area. Although the sample size was small, the participants were obstetricians with vast knowledge and expertise in obstetrics, which enabled them to provide rich, comprehensive insights into the research topic. Therefore, the adequacy of the data was achieved with fewer participants. The participants’ expertise in obstetrics ensured the emergence of key themes and patterns consistently across interviews, which confirmed data saturation despite the small sample size. A major limitation is that the study was conducted in the academic hospitals, an obstetrician-led environment based on the complexity of obstetric conditions being managed at that level of care. The autonomous role and responsibilities of the midwife specialists are more apparent in the lower levels of care such as primary health care clinics, midwife-led obstetric units and district hospitals. These are facilities where obstetricians do not render direct health care services collaboratively with the midwife specialists. Therefore, the obstetricians’ perspectives are limited to midwife specialists’ role in academic hospitals, and findings cannot be generalised to lower-level facilities.

The study forms part of a doctoral study in which midwife specialists’ practice was explored and described. The obstetricians described midwife specialists’ practice from their own experiences and perceptions, which provides a varying perspective.

## Conclusion

A qualitative and descriptive research design described midwife specialists’ limited practice in the public health sectors as perceived by obstetricians. The findings of our study have shown sub-optimal practice in terms of utilisation of MS knowledge and skills in clinical practice. The misalignment between the SOP of midwives and the expanded knowledge and skills of the MS brought about limited practice. The study suggests a need for the SANC and the National Department of Health to review the SOP to provide the specific MS roles and responsibilities. The revision of the SOP will reduce the midwife specialists’ susceptibility to litigations and defensive practice, evidenced by over-reliance on obstetricians. These findings may influence the revision of nursing and midwifery education to provide training aligned with the ICM competencies and the SOP of midwife specialists. The alignment of midwife specialists’ training and practice regulation may potentially eliminate the fear of litigation, ultimately leading to midwives’ specialists’ optimal practice.

### Recommendations and implications

The study’s findings highlight critical implications for midwifery practice, education, research and policy to ensure recognition and enable the autonomous and independent role of the midwife specialists in South Africa. In midwifery practice, the midwife specialists are reminded of their critical role as primary caregivers and collaborators in maternal and neonatal healthcare. This involves midwife specialists assuming their roles and demonstrating their autonomous and independent function.

From an educational perspective, there is a need to re-empower midwives to improve their knowledge and skills set that might have been potentially lost as they were defensive in their practice. The re-empowerment of midwife specialists will revive their clinical competence, which will in turn heighten their professional confidence. Furthermore, there is a need to reintroduce the midwife specialists as the empowered practitioner and a specialist to the interprofessional collaborators, especially the obstetricians to change their current perception of the midwife specialist.

In the research context, a comparative study between South African midwife specialist and midwife specialist in other countries and how they navigate their roles in the clinical settings is recommended. Based on the obstetricians’ perspectives of the midwife specialists in South Africa, there is a need for a study on IPC between the midwife specialists and obstetricians. A study on the development of recommendations for an SOP for MS to inform a regulation is recommended.

Policy recommendations call for the revision of the SOP to explicitly define the midwife specialists’ roles and responsibilities. The clarification of roles and responsibilities will ensure clear demarcation between registered midwife and midwives specialists. In addition, the presence of the SOP may eliminate the current defensive practice, which hinders the autonomous and independent functions necessary to improve the patients’ outcomes.
